# Supplementary material for: Federated learning for the pathogenicity annotation of genetic variants in multi-site clinical settings
Source: Bioinformatics. 2025 Sep 19;41(10):btaf523. doi: 10.1093/bioinformatics/btaf523 (PMC12517337; doi:10.1093/bioinformatics/btaf523)
Supplement: btaf523_Supplementary_Data [file btaf523_supplementary_data.zip › SupplementaryMaterial_Bioinformatics_Revised.pdf]

Federated Learning for the pathogenicity annotation of genetic variants in multi-site clinical settings.

Nigreisy Montalvo [1], Francisco Requena [2], Emidio Capriotti[3] & Antonio Rausell [1,4,*]

(1) Université Paris Cité, INSERM UMR1163, Imagine Institute, Clinical Bioinformatics Laboratory, Paris, F-75006, France.
(2) Department of Physiology and Biophysics, Weill Cornell Medicine, Institute for Computational Biomedicine, Englander Institute for Precision Medicine, New York, NY, 10021, USA
(3) Department of Pharmacy and Biotechnology (FaBiT), University of Bologna, Bologna 40126, Italy.
(4) AP-HP, Necker Hospital for Sick Children, Fédération de Génétique et Médecine Génomique, Service de Médecine Génomique des Maladies Rares, Paris, F-75015, France.

* Correspondence to: antonio.rausell@institutimagine.org

Supplementary data

Supplementary Figures

Supplementary Figure 1

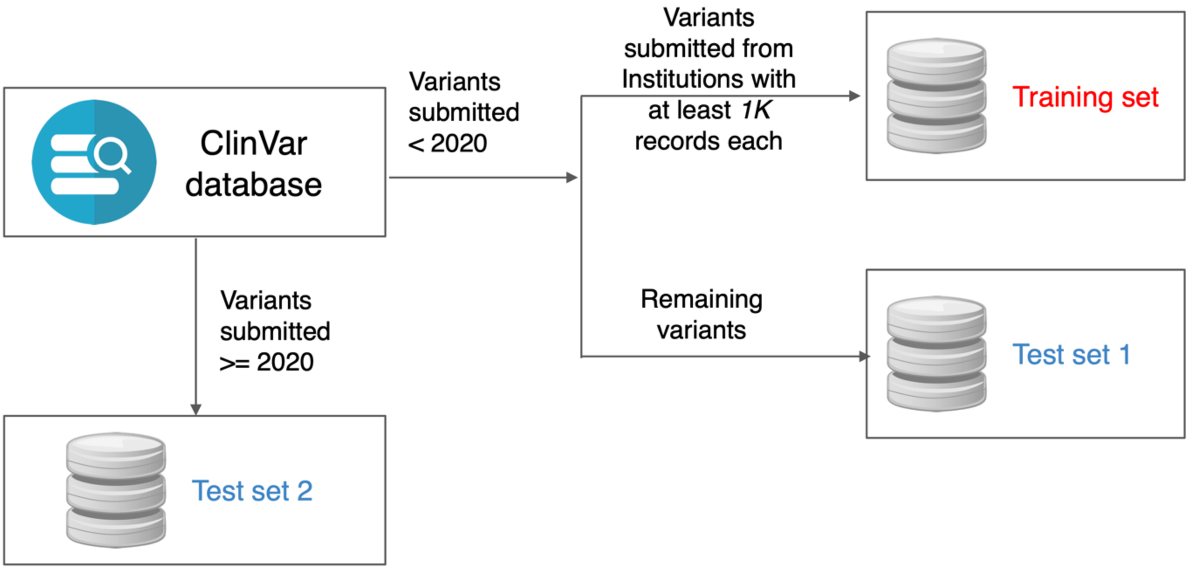

Supplementary Figure 1: Workflow followed to construct the training and test sets of coding SNV variants used in this study. ClinVar variants were first split into two non-overlapping subsets based on the submission date. Variants reported before January 1st, 2020 were further divided into two non-overlapping subsets: (i) those submitted by major contributors (defined as institutions contributing at least 1K variants each), which constitute the set used for training, either in a centralized or federated learning setting; and (ii) the remaining variants (i.e., submitted by non-major contributors), referred to in the text as Test Set 1. Finally, variants reported after January 1st, 2020 were reserved for creating a second test set, referred to in the text as Test Set 2. An analogous process was independently followed for non-coding SNVs and CNVs (see details in Methods).

Supplementary Figure 2

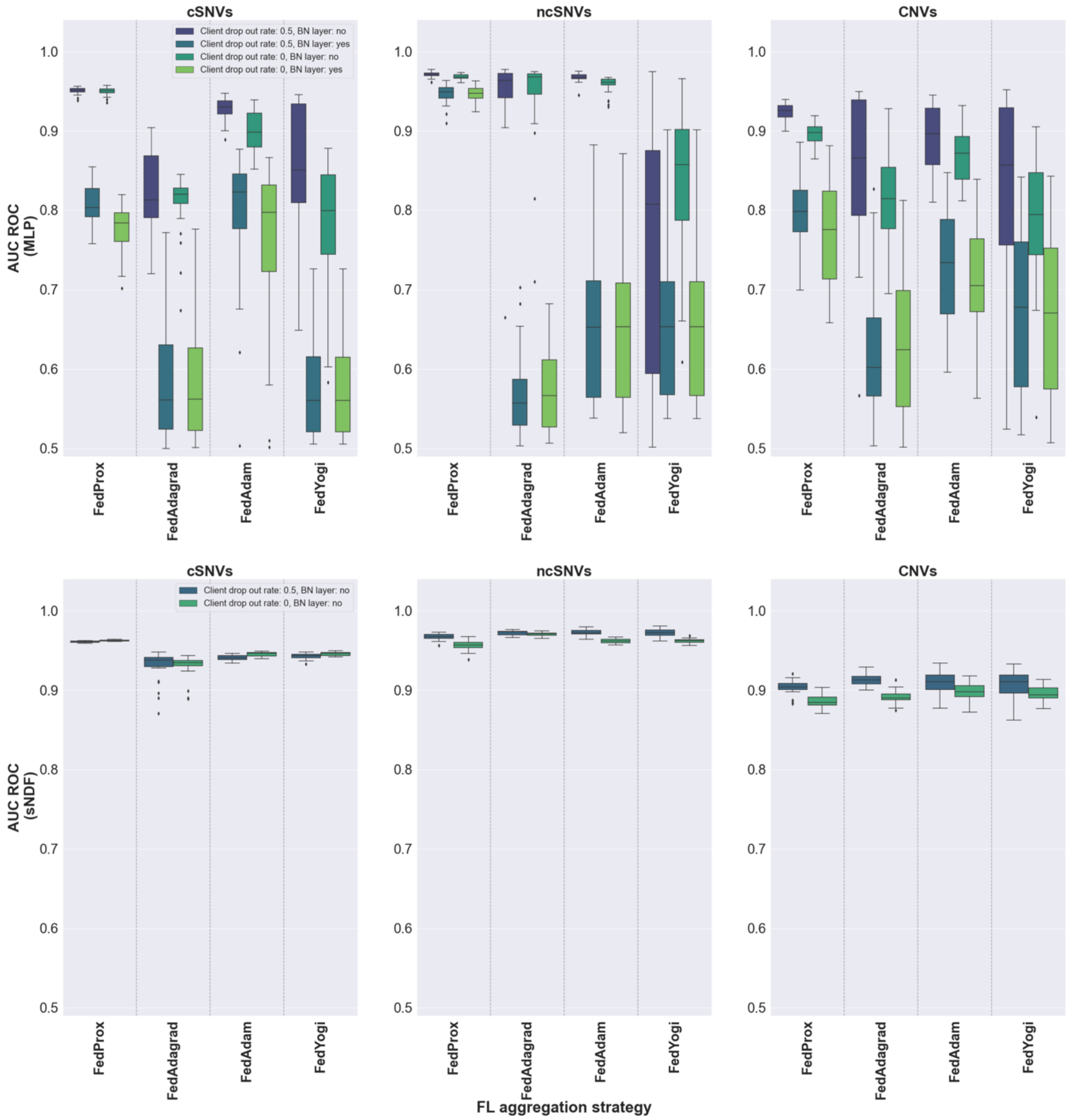

Supplementary Figure 2: AUC ROC values obtained through cross-validation for different FL aggregation strategies across three types of genetic variants: coding SNVs (left), non-coding SNVs (middle), and CNVs (right panels). FL aggregation strategies considered are indicated in the x-axis: FedProx, FedAdagrad, FedAdam, and FedYogi. Panels in the first row present the performance of such FL aggregation strategies when applied to the MLP model, with varying FL client participation rates of 50% and 100%, and considering the inclusion or exclusion of local batch normalization layers as indicated in the legend (as shown in the inset in the top-left panel). The second row displays the performance of the FL strategies based on sNDF models, also considering FL client rates of 50% and 100%. Boxplots in the panels represent the distribution of AUC ROC values obtained upon 30 different random seeds for model weight initialization. A perfect classifier would achieve an AUC ROC value equal to 1, while a random classifier would achieve an AUC ROC value of 0.5. Therefore, for all panels, a higher AUC indicates a better performance.

Supplementary Figure 3

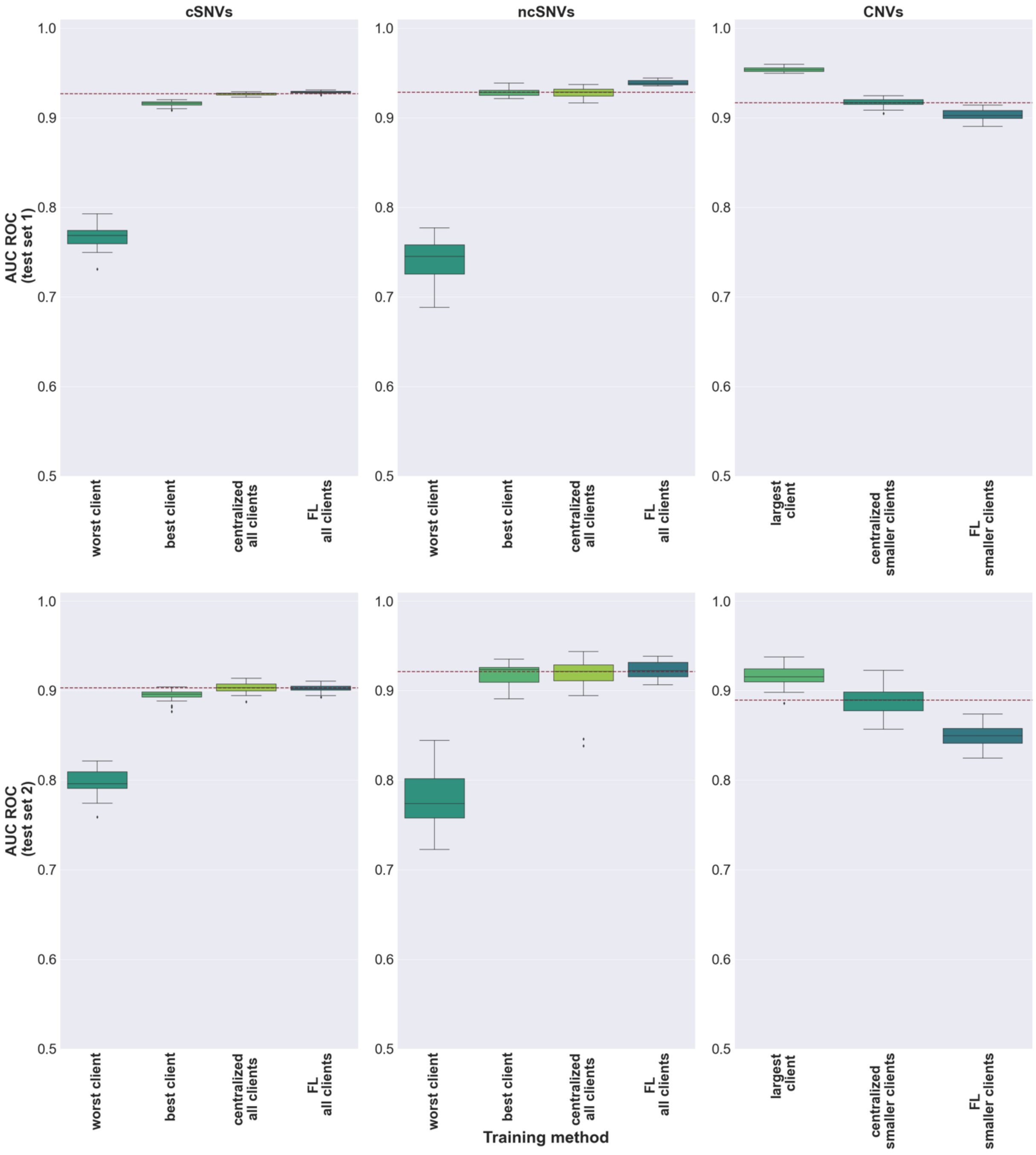

Supplementary Figure 3. Performance of local, centralized, and federated sNDF models on two independent test sets. Top and bottom panels show, respectively, the results on the first and second independent sets (**Methods**). In the case of coding SNVs and non-coding SNVs, the performance of the worst and best individual client models as well as the pooled-clients models are displayed. The performances of the remaining client models are reported in **Supplementary Tables 7-9**. In the case of CNVs, the performance of the largest client was compared against the centralized and federated learning models of the smaller clients. Boxplots in the panels represent the distribution of AUC ROC values obtained upon 30 different random seeds for model weight initialization. To ease comparison across models, a dotted red line represents the median value obtained for the centralized models.

Supplementary Figure 4

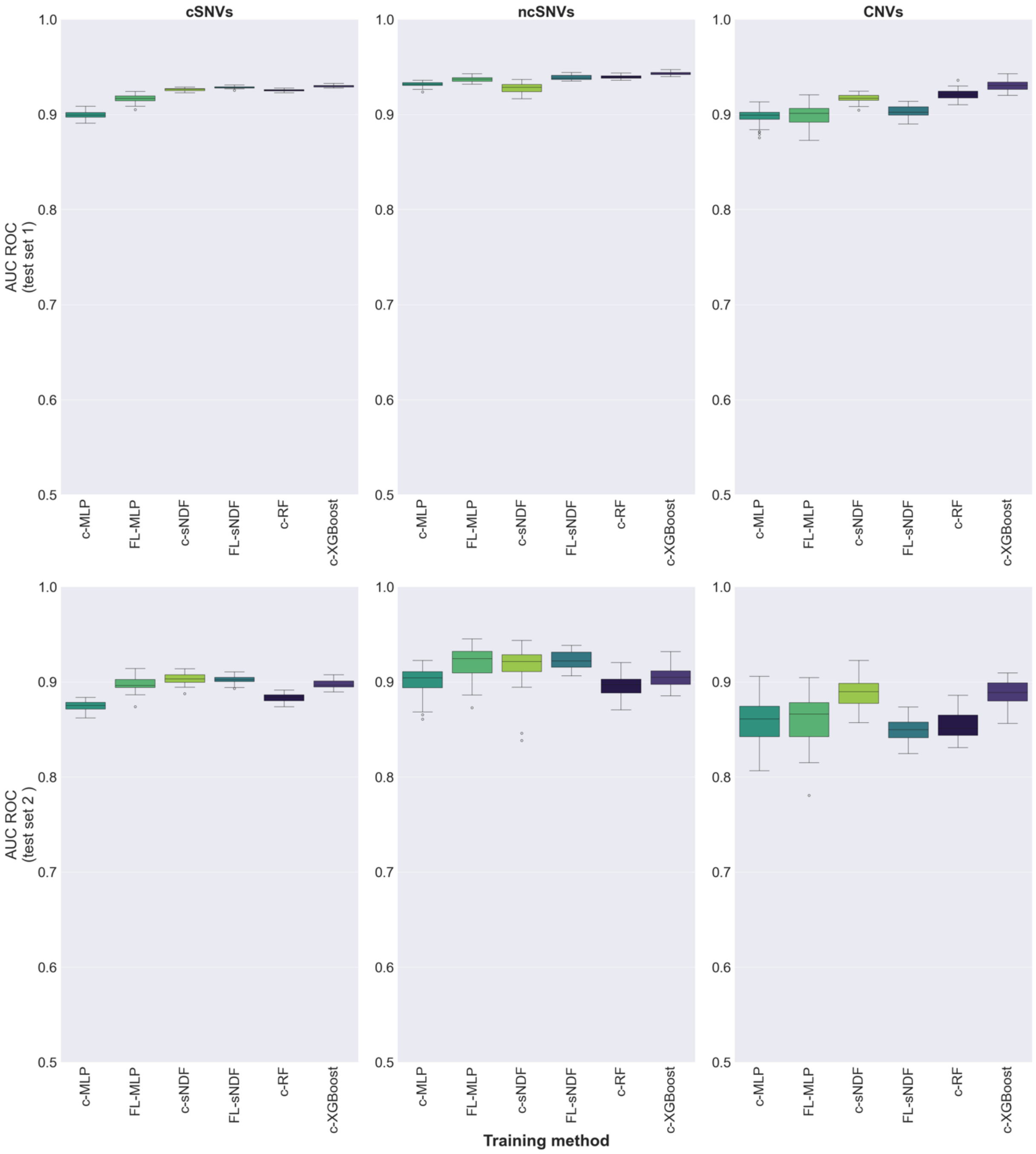

Supplementary Figure 4. Performance of non-differentiable centralized models and of centralized and federated MLP and sNDF models on two independent test sets. The performance of two non-differentiable learning algorithms, random forest (RF) and XGBoost, trained on the centralized datasets is shown together with that of the centralized and federated MLP and sNDF models. Top and bottom panels show, respectively, the results on the first and second independent sets (Methods) for coding SNVs (left), non-coding SNVs (middle) and CNVs (right panels). Boxplots in the panels represent the distribution of AUC ROC values obtained upon 30 different random seeds for model weight initialization.

Supplementary Figure 5

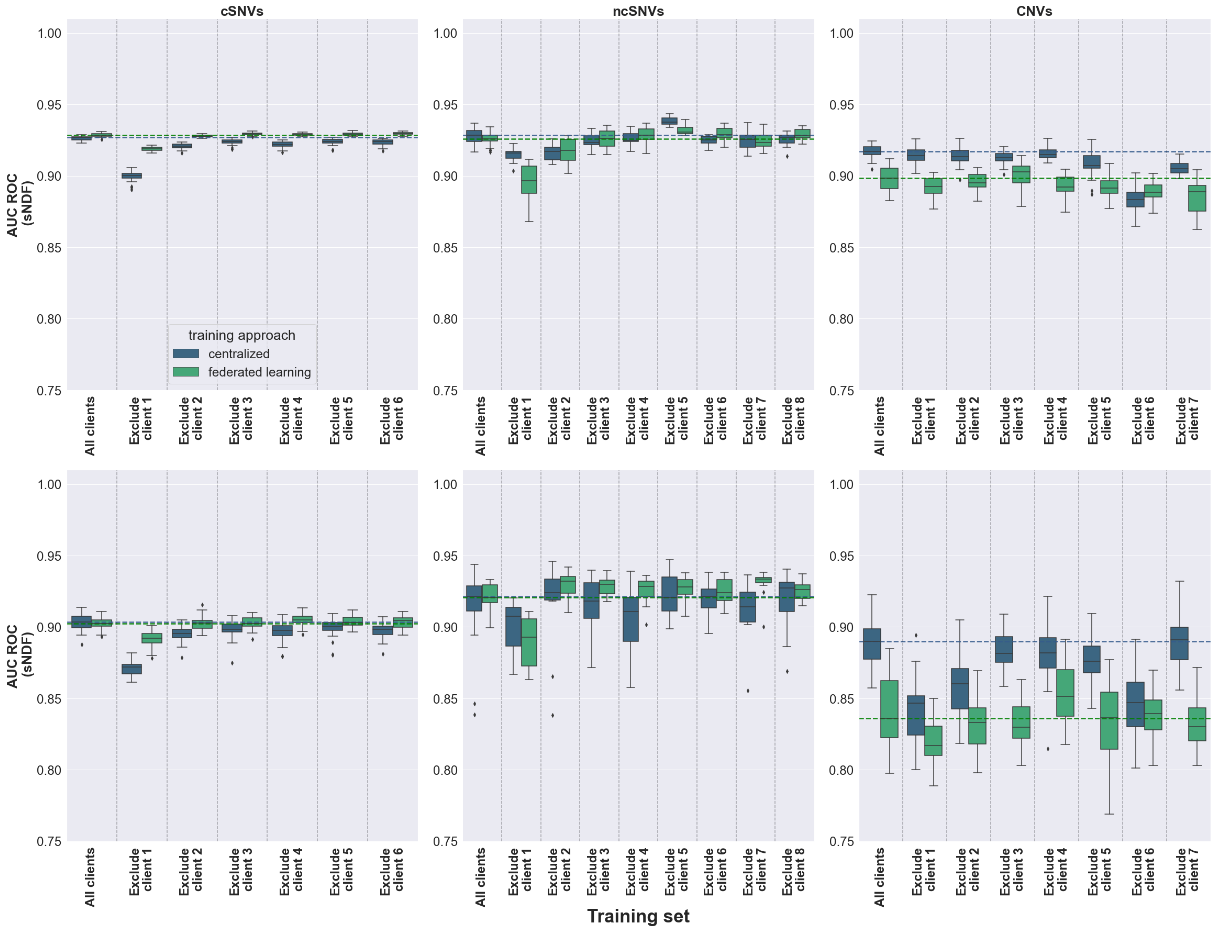

Supplementary Figure 5. Performance of centralized, and federated sNDF models upon client dropouts on two independent test sets. Top and bottom panels show, respectively, the results obtained on the first and second independent tests (**Methods**). Boxplots in the panels represent the distribution of AUC ROC values obtained upon 30 different random seeds for model weight initialization. Centralized and FL models are colored in blue and green, respectively. Each panel represents the AUC ROC values obtained considering all clients, as well as excluding one client at a time. To ease comparison across models, dotted lines represent the median values obtained for the centralized (blue) and federated (green) models considering all clients.

Supplementary Figure 6

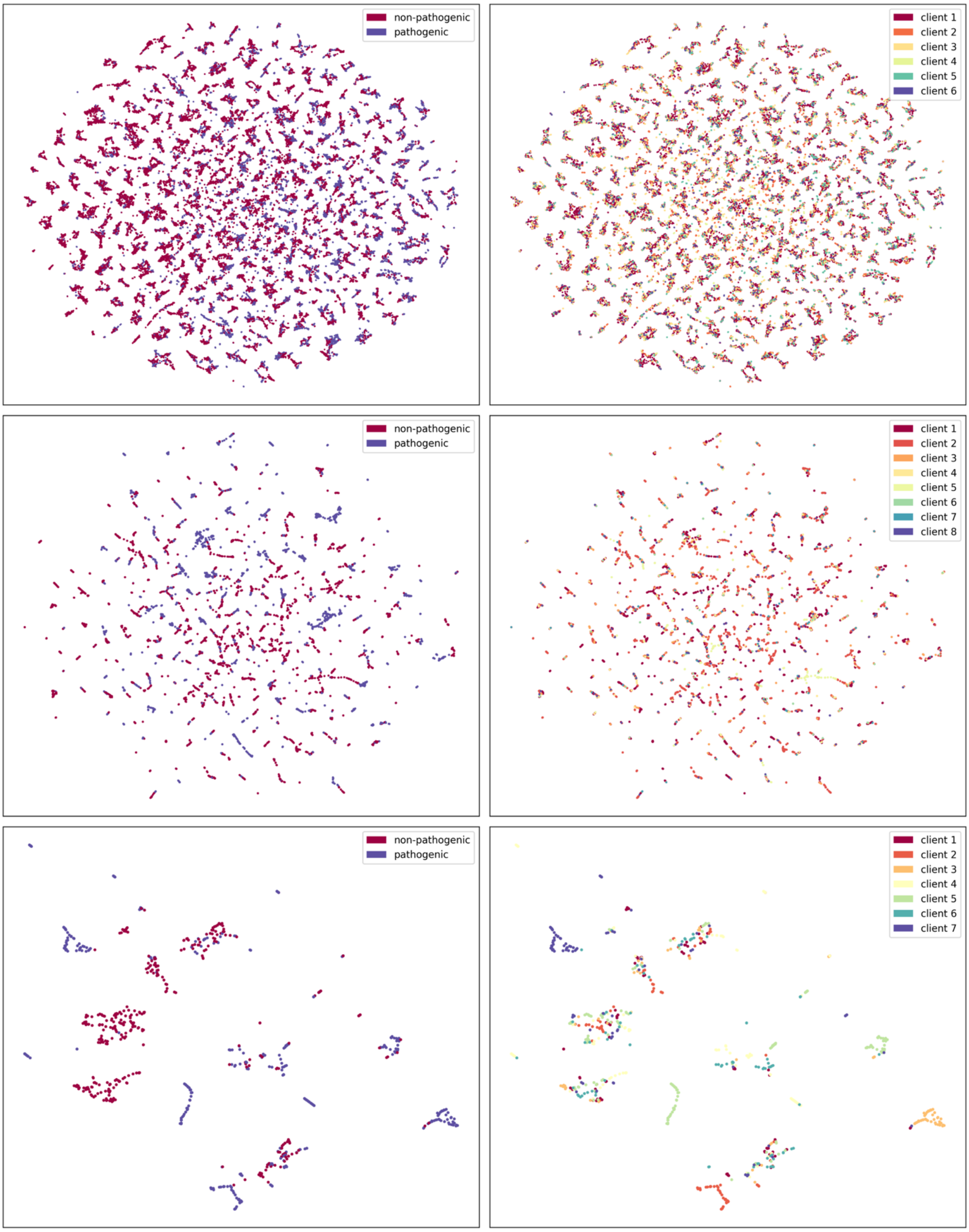

Supplementary Figure 6. Variant distribution in an unsupervised low-dimensional UMAP representation obtained from the variants feature matrices. Coding SNVs, non-coding SNVs and CNVs are represented in top, middle, and bottom panels, respectively. Panels on the left represent variants colored according to their clinical label: pathogenic (blue) or benign (red). Panels on the right are colored according to their submitter institution (see **Supplementary Tables 2-5**).

Supplementary Tables

Variant type	Coding SNVs	Non-coding SNVs	Deletion CNVs
Number of features	60	60	38
Model comparisons	Single clients vs FL of all clients vs CDS of all clients	Single clients vs FL of all clients vs CDS of all clients	Largest client vs FL of smaller clients vs CDS of smaller clients
Learning algorithm	MLP and sNDF		
FL aggregation strategy	FedProx, FedAdam, FedAdagrad, FedYogi		
Client rate	50% and 100%		
Evaluation	Collaborative Cross-Validation (CCV)<br>Two held-out test sets		
Metric	AUC ROC		

Supplementary Table 1: Overview of the collaborative training experiments. The table summarizes the key configurations explored in the collaborative training of machine learning models for the clinical assessment of coding SNVs, non-coding SNVs, and deletion CNVs. It details the number of features used for variant annotation, the models trained, namely Multilayer Perceptron (MLP) and Shallow Neural Decision Forest (sNDF), the baseline models compared for each case study, the federated learning (FL) aggregation strategies employed, and the client participation rates during local training in each round of federated training. Additionally, it outlines the datasets used for evaluation and the evaluation metric applied, which is the area under the ROC curve (AUC ROC).

Number id	Institution	Headquarters	Dataset size (original)	Pathogenic	Benign	Dataset size (after random under-sampling)
1	Invitae	San Francisco, California, United States	30,815	8114	22,701	16,228
2	GeneDx	Gaithersburg, Maryland, United States	6177	3945	2232	4464
3	Illumina Laboratory Services, Illumina	San Diego, California, United States	4680	123	4557	246
4	EGL Genetic Diagnostic, Eurofin Clinical Diagnostics	Tucker, Georgia, United States	1940	1143	797	1594
5	Ambry Genetics	Aliso Viejo, California, United States	2,527	1629	898	1,796
6	Laboratory for Molecular Medicine, Partners Healthcare, Personalized Medicine	Boston, Massachusetts, United States	1417	362	1055	724

Supplementary Table 2: Details of clients in the multi-institutional coding single nucleotide variants dataset. The table provides detailed information about the institutions included in the multi-institutional coding single nucleotide variants dataset derived from the ClinVar database. It lists the names and locations of the contributing institutions, along with the original dataset size and the adjusted size following random under-sampling of the majority class. Additionally, it specifies the number of pathogenic and benign samples associated with each institution. The first column indicates the client id used along the manuscript for this variant type.

Number id	Institution	Headquarters	Dataset size (original)	Pathogenic	Benign	Data size (after random under-sampling)
1	GeneDx	Gaithersburg, Maryland, United States	2,288	560	1,728	1,120
2	Invitae	San Francisco, California, United States	1,735	965	770	1,540
3	EGL Genetic Diagnostics, Eurofins Clinical Diagnostics	Tucker, Georgia, United States	357	288	69	138
4	Laboratory for Molecular Medicine, Partners Healthcare Personalized Medicine	Boston, Massachusetts, United States	136	60	76	120
5	Wong Mito Lab, Molecular and Human Genetics, Baylor College of Medicine	Houston, Texas, United States	176	52	124	104
6	Athena Diagnostics	Marlborough, Massachusetts, United States	101	64	37	74
7	Integrated Genetics/ Laboratory Corporation of America, LabCorp	Westborough, Massachusetts, United States	108	73	25	50
8	ARUP Laboratories, Molecular Genetics and Genomic	Salt Lake City, Utah, United States	126	33	93	66

Supplementary Table 3: Details of clients in the multi-institutional non-coding single nucleotide variants dataset. The table provides detailed information about the institutions included in the multi-institutional non-coding single nucleotide variants dataset derived from the ClinVar database. It lists the names and locations of the contributing institutions, along with the original dataset size and the adjusted size following random under-sampling of the majority class. Additionally, it specifies the number of pathogenic and benign samples associated with each institution. The first column indicates the client id used along the manuscript for this variant type.

Number	Institution	Headquarters	Dataset size	Pathogenic	Benign
1	Invitae	San Francisco, California, United States	6,244	3,122	3,122
2	Laboratory for Molecular Medicine, Partners Healthcare, Personalized Medicine	Boston, Massachusetts, United States	82	41	41
3	Lupski Lab, Baylor-Hopkins CMG, Baylor College of Medicine	Houston, Texas, United States	88	44	44
4	Consortium of Investigators of Modifiers of BRCA ½ (CIMBA), c/o University of Cambridge	Cambridge, United Kingdom	86	43	43
5	Women's Health and Genetics/Laboratory Corporation of America, LabCorp	Westborough, Massachusetts, United States	110	55	55
6	International Society for Gastrointestinal Hereditary Tumours (InSiGHT)	London, United Kingdom	150	75	75
7	Baylor Genetics	Houston, Texas, United States	86	43	43
8	LDLR-LOVD, British Heart Foundation	London, United Kingdom	90	45	45

Supplementary Table 4: Details of clients in the multi-institutional deletion copy number variants dataset. The table provides detailed information about the institutions included in the multi-institutional deletion copy number variants dataset derived from the ClinVar database. It lists the names and locations of the contributing institutions, along with the original dataset size and the number of pathogenic and benign samples associated with each institution. The first column indicates the client id used along the manuscript for this variant type.

Supplementary Table 5. Hyperparameters retained for the centralized and federated models for the pathogenicity annotation of coding SNVs, non-coding SNVs, and CNVs. See Excel file attached.

Genetic variant type	No. clients	Training set size	Test set 1 size	Test set 2 size
**Coding SNVs**	6	79,556	10,378	2,838
**Non-coding SNVs**	8	11,396	5,534	472
**CNVs**	8	6,936	682	96

Supplementary Table 6: Size of multi-institutional datasets and independent test sets per genetic variant type. The table summarize the number of institutions, the total number of genetic variants present in the multi-institutional training dataset, and the number of variants in the two independent test sets for each variant type.

**Model**	**MLP**		**sNDF**	
	**Test set 1**	**Test set 2**	**Test set 1**	**Test set 2**
**Client 1**	0.89	0.87	0.92	0.90
**Client 2**	0.88	0.86	0.88	0.86
**Client 3**	0.73	0.76	0.77	0.80
**Client 4**	0.86	0.85	0.85	0.85
**Client 5**	0.85	0.86	0.84	0.86
**Client 6**	0.79	0.80	0.84	0.85
**CDS**	0.90	0.88	0.93	0.90
**FL**	0.92	0.90	0.93	0.90

Supplementary Table 7. Performance of local, centralized and federated models for the clinical assessment of coding single nucleotide variants. The table presents the mean area under the ROC curve (AUC ROC) values for the clinical assessment of coding single nucleotide variants, comparing local, centralized, and federated models. The performance of the Multilayer Perceptron (MLP) and Shallow Neural Decision Forest (sNDF) models is reported for each approach: local (denoted as client i), centralized (CDS), and federated learning (FL), evaluated across two independent test sets.

Model	MLP		sNDF	
	**Test set 1**	**Test set 2**	**Test set 1**	**Test set 2**
**Client 1**	0.94	0.94	0.93	0.93
**Client 2**	0.93	0.90	0.93	0.90
**Client 3**	0.85	0.86	0.86	0.87
**Client 4**	0.84	0.81	0.86	0.80
**Client 6**	0.80	0.80	0.85	0.86
**Client 7**	0.69	0.70	0.75	0.78
**Client 8**	0.75	0.76	0.80	0.82
**CDS**	0.93	0.90	0.93	0.93
**FL**	0.94	0.93	0.94	0.93

Supplementary Table 8: Performance of local, centralized and federated models for the clinical assessment of non-coding single nucleotide variants. The table presents the mean area under the ROC curve (AUC) values for the clinical assessment of non-coding single nucleotide variants, comparing local, centralized, and federated models. The performance of the Multilayer Perceptron (MLP) and Shallow Neural Decision Forest (sNDF) models is reported for each approach: local (denoted as client i), centralized (CDS), and federated learning (FL), evaluated across two independent test sets. The results of local model corresponding to client 5 are not listed, since this institution did not have variants found on each chromosome.

**Model**	**MLP**		**sNDF**	
	Test set 1	Test set 2	Test set 1	Test set 2
**Largest client**	0.96	0.92	0.96	0.92
**CDS**	0.90	0.87	0.92	0.89
**FL**	0.90	0.87	0.90	0.86

Supplementary Table 9: Performance of largest client, centralized, and federated models of smaller clients for the clinical assessment of deletion copy number variants. The table presents the mean area under the ROC curve (AUC ROC) values for the clinical assessment of deletion copy number variants, comparing the performance of models trained with the largest client, centralized models, and federated models of smaller clients. The table reports the results for the Multilayer Perceptron (MLP) and Shallow Neural Decision Forest (sNDF) models across three approaches: the largest client (Invitae), centralized (CDS), and federated learning (FL), evaluated on two independent test sets**.**

Variant type	MLP				sNDF			
	**Test set 1**		**Test set 2**		**Test set 1**		**Test set 2**	
	**Stat.**	**p-value**	**Stat.**	**p-value**	**Stat.**	**p-value**	**Stat.**	**p-value**
**cSNVs**	-6.59	4.28e−11	-6.40	1.53e−10	-5.04	4.62e−07	0.84	0.40
**ncSNVs**	-5.63	1.77e−08	-4.33	1.47e−05	-6.51	7.03e−11	-0.77	0.44
**CNVs**	-0.99	0.32	-0.62	0.53	6.28	3.31e−10	6.20	5.57e−10

Supplementary Table 10: Wilcoxon rank-sum test results for centralized and federated models in the clinical assessment of genetic variants. The table presents the results of Wilcoxon rank-sum test, comparing the mean area under the curve ROC obtained by the best-performing centralized and federated learning models across two independent test sets. The Wilcoxon rank-sum statistic and corresponding p-values are reported for each type of genetic variant: coding single nucleotide variant (cSNV), non-coding single nucleotide variant (ncSNV), and deletion copy number variant (CNV). Results are shown for both the Multilayer Perceptron (MLP) and Shallow Neural Decision Forest (sNDF) models.

	Coding SNVs		Non-coding SNVs		CNVs	
**Model**	**Test set 1**	**Test set 2**	**Test set 1**	**Test set 2**	**Test set 1**	**Test set 2**
**CDS-MLP**	0.90	0.88	0.93	0.90	0.90	0.87
**CDS-sNDF**	0.93	0.90	0.93	0.93	0.92	0.89
**CDS-RF**	0.93	0.88	0.94	0.90	0.92	0.86
**CDS-XGBoost**	0.93	0.90	0.94	0.90	0.93	0.89
**FL-MLP**	0.92	0.90	0.94	0.93	0.90	0.87
**FL-sNDF**	0.93	0.90	0.94	0.93	0.90	0.86

Supplementary Table 11. Performance of non-differentiable centralized models and of centralized and federated MLP and sNDF models on two independent test sets. The table presents the mean area under the ROC curve (AUC ROC) values for the clinical assessment of the different variant types (coding single nucleotide variants, non-coding single nucleotide variants and CNV), comparing non-differentiable centralized (CDS) models - Radom Forest (RF) and XGBoost- and centralized and federated (FL) MLP and sNDF models across two independent test sets for each variant type.

		MLP				sNDF			
Variant type		Test set 1		Test set 2		Test set 1		Test set 2	
	Training set	Stat.	p-value	Stat.	p-value	Stat.	p-value	Stat.	p-value
**cSNVs**	Dropping client 1	4.54	5.66e-06	3.81	1.36e-04	6.65	2.87e-11	6.65	2.87e-11
	Dropping client 2	6.65	2.87e-11	6.64	3.18e-11	6.57	4.73e-11	4.70	2.58e-06
	Dropping client 3	6.65	2.87e-11	6.47	9.45e-11	4.39	1.13e-05	3.08	2.00e-03
	Dropping client 4	6.65	2.87e-11	6.53	6.37e-11	6.23	4.40e-10	3.70	2.19e-04
	Dropping client 5	6.65	2.87e-11	6.37	1.86e-10	4.54	5.66e-06	2.29	0.02
	Dropping client 6	6.65	2.87e-11	6.61	3.88e-11	4.91	9.18e-07	3.36	7.91e-04
**ncSNVs**	Dropping client 1	6.65	2.87e-11	6.05	1.47e-09	4.59	4.43e-06	2.94	3.26e-03
	Dropping client 2	6.65	2.87e-11	5.81	6.24e-09	4.32	1.52e-05	-0.62	0.54
	Dropping client 3	6.64	3.18e-11	4.73	2.15e-06	2.03	0.04	0.21	0.84
	Dropping client 4	6.65	2.87e-11	6.34	2.15e-10	0.94	0.35	1.50	0.13
	Dropping client 5	6.65	2.87e-11	4.95	7.31e-07	-4.71	2.50e-06	-0.19	0.85
	Dropping client 6	6.65	2.87e-11	4.28	1.81e-05	2.09	0.04	0.17	0.86
	Dropping client 7	6.65	2.87e-11	5.23	1.66e-07	1.56	0.12	1.40	0.16
	Dropping client 8	6.65	2.87e-11	5.23	1.66e-07	1.59	0.11	-0.22	0.83
**CNVs**	Dropping client 1	2.61	8.87e-03	5.44	5.31e-08	2.59	0.01	6.20	5.57e-10
	Dropping client 2	0.04	0.96	1.36	0.17	2.70	0.01	5.12	3.01e-07
	Dropping client 3	1.43	0.15	-1.52	0.13	3.78	1.58e-04	1.42	0.16
	Dropping client 4	1.23	0.26	5.49	3.96e-08	1.75	0.08	1.43	0.15
	Dropping client 5	1.06	0.29	-1.98	0.05	4.14	3.47e-05	2.71	0.01
	Dropping client 6	6.26	4.01e-10	0.21	0.83	6.65	2.87e-11	5.97	2.43e-09
	Dropping client 7	5.01	5.39e-07	-3.80	1.44e-04	6.15	7.73e-10	-0.31	0.75

Supplementary Table 12: Wilcoxon rank-sum test comparing centralized models before and after dropping a client from the training set. The table presents the results of the Wilcoxon rank-sum tests comparing the mean area under the curve ROC of the best-performing centralized model trained across of all clients and those trained after dropping a given client i. across the two independent test sets. The Wilcoxon rank-sum statistic and corresponding p-values are reported for each type of genetic variant: coding single nucleotide variant (cSNV), non-coding single nucleotide variant (ncSNV), and deletion copy number variant (CNV). Results are shown for both the Multilayer Perceptron (MLP) and Shallow Neural Decision Forest (sNDF) models.

		MLP				sNDF			
Variant type		**Test set 1**		**Test set 2**		**Test set 1**		**Test set 2**	
	**Training set**	**Stat.**	**p-value**	**Stat.**	**p-value**	**Stat.**	**p-value**	**Stat.**	**p-value**
**cSNVs**	Dropping client 1	6.21	5.32e-10	4.92	8.51e-07	6.65	2.87e-11	5.91	3.34e-09
	Dropping client 2	1.98	0.23	1.33	0.18	2.42	0.02	-0.27	0.79
	Dropping client 3	-0.41	0.68	-0.22	0.83	-2.61	0.01	-0.41	0.68
	Dropping client 4	0.38	0.70	-0.17	0.86	-1.82	0.07	-2.94	3.25e-03
	Dropping client 5	-0.06	0.95	0.80	0.42	-1.33	0.18	-1.27	0.20
	Dropping client 6	-0.34	0.73	-0.21	0.83	-3.46	5.41e-04	-1.12	0.26
**ncSNVs**	Dropping client 1	5.26	1.42e-07	1.90	0.06	4.86	1.20e-06	4.56	5.09e-06
	Dropping client 2	5.92	3.06e-09	2.76	0.01	2.82	4.73e-03	-2.47	0.01
	Dropping client 3	0.38	0.70	0.94	0.34	0.03	0.98	-2.32	0.02
	Dropping client 4	0.04	0.96	-0.93	0.35	-1.29	0.19	-1.50	0.13
	Dropping client 5	-0.96	0.34	-0.87	0.38	-3.53	4.14e-04	-2.04	0.04
	Dropping client 6	0.89	0.37	-0.45	0.65	-1.85	0.06	-1.27	0.21
	Dropping client 7	-0.80	0.42	-0.66	0.51	1.26	0.21	-3.41	6.41e-04
	Dropping client 8	0.24	0.81	-1.29	0.20	-1.70	0.09	-1.06	0.29
**CNVs**	Dropping client 1	0.50	0.62	2.65	8.14e-03	2.74	0.01	3.49	4.71e-04
	Dropping client 2	-0.81	0.42	-0.55	0.58	1.28	0.20	1.22	0.21
	Dropping client 3	-3.56	3.67e-04	0.62	0.53	-1.47	0.13	1.25	0.21
	Dropping client 4	1.29	0.20	0.59	0.55	2.23	0.02	-2.29	0.02
	Dropping client 5	-0.89	0.38	1.18	0.24	2.96	3.11e-03	0.98	0.33
	Dropping client 6	3.58	3.46e-04	1.79	0.07	3.70	2.18e-04	0.03	0.97
	Dropping client 7	1.11	0.27	-3.17	1.51	4.05	5.10e-05	1.25	0.21

Table 13: Wilcoxon rank-sum test comparing federated learning models before and after dropping a client from the training set. This table presents the results of Wilcoxon rank-sum test, comparing the mean area under the curve ROC of the best-performing federated learning model trained across of clients considered initially in the simulations, and after dropping client i. across the two independent test sets. The Wilcoxon rank-sum statistic and corresponding p-values are reported for each type of genetic variant: coding single nucleotide variant (cSNV), non-coding single nucleotide variant (ncSNV), and deletion copy number variant (CNV). Results are shown for both the Multilayer Perceptron (MLP) and Shallow Neural Decision Forest (sNDF) models.

Variant type	MLP				sNDF			
	**Test set 1**		**Test set 2**		**Test set 1**		**Test set 2**	
	**Stat.**	**p-value**	**Stat.**	**p-value**	**Stat.**	**p-value**	**Stat.**	**p-value**
**cSNVs**	0.93	6.03e−303	0.94	6.88e−298	0.95	2.16e−132	0.96	6.45e−307
**ncSNVs**	0.93	1.78e−277	0.94	3.26e−228	0.94	4.62e−145	0.97	4.78e−291
**CNVs**	0.88	3.32e−224	0.86	1.89e−30	0.87	4.53e−213	0.82	1.36e−25

Supplementary Table 14. Spearman's rank correlation test results for centralized and federated models in the clinical assessment of genetic variants. The table presents the results of Spearman's rank correlation test, comparing the pathogenicity predictions of the best-performing centralized and federated learning models across two independent test sets. The Spearman's rank correlation coefficient and the associated p-value are reported for each genetic variant type: coding single nucleotide variants (cSNVs), non-coding single nucleotide variants (ncSNVs), and deletion copy number variants (CNVs). Results are shown for both the Multilayer Perceptron (MLP) and Shallow Neural Decision Forest (sNDF) models.
